# The impact of biliary stents on the diagnostic yield of endoscopic ultrasound‐guided fine needle aspiration for solid pancreatic lesions: A single‐center retrospective study and meta‐analysis

**DOI:** 10.1002/deo2.250

**Published:** 2023-07-10

**Authors:** Go Endo, Kazunaga Ishigaki, Tsuyoshi Hamada, Yousuke Nakai, Kota Ishida, Kohei Kurihara, Shuichi Tange, Shinya Takaoka, Yurie Tokito, Yukari Suzuki, Hiroki Oyama, Sachiko Kanai, Tatsunori Suzuki, Tatsuya Sato, Ryunosuke Hakuta, Tomotaka Saito, Naminatsu Takahara, Mitsuhiro Fujishiro

**Affiliations:** ^1^ Department of Gastroenterology Graduate School of Medicine the University of Tokyo Tokyo Japan; ^2^ Department of Chemotherapy the University of Tokyo Hospital Tokyo Japan; ^3^ Department of Endoscopy and Endoscopic Surgery the University of Tokyo Hospital Tokyo Japan

**Keywords:** cholangiopancreatography, endoscopic retrograde, endoscopic ultrasound‐guided fine needle aspiration, jaundice, obstructive, pancreatic neoplasms, stents

## Abstract

**Background:**

Endoscopic ultrasound‐guided fine needle aspiration (EUS‐FNA) is widely used for the pathological diagnosis of solid pancreatic lesions but in cases with obstructive jaundice, transpapillary sampling can be performed during endoscopic retrograde cholangiopancreatography with transpapillary biliary stent placement. Thus, it is still controversial whether EUS‐FNA should be performed prior to endoscopic retrograde cholangiopancreatography with biliary stent placement or only after negative transpapillary sampling.

**Methods:**

The accuracy, sensitivity, and specificity of EUS‐FNA for solid pancreatic lesions with or without indwelling biliary stents were retrospectively studied in patients undergoing EUS‐FNA between January 2017 and December 2021. We also conducted a meta‐analysis including our data to compare the accuracy and sensitivity of EUS‐FNA with or without biliary stents.

**Results:**

A total of 509 patients (40 with biliary stents and 469 without biliary stents) were included. The accuracy (77.5% vs. 94.5%, *p* < 0.001) and sensitivity (71.0% vs. 91.7%, *p* < 0.001) were lower in EUS‐FNA with biliary stents. A meta‐analysis confirmed that accuracy (odds ratio [OR] of 0.43, 95% confidence interval [CI] 0.29–0.62, *p* < 0.001) and sensitivity (OR of 0.46, 95% CI 0.33–0.64, *p* < 0.001) were lower in EUS‐FNA with biliary stents. There were no statistically significant differences between plastic stents and self‐expandable metallic stents for accuracy or sensitivity.

**Conclusions:**

The presence of biliary stents had a negative impact on the diagnostic performance of EUS‐FNA, and EUS‐FNA prior to endoscopic retrograde cholangiopancreatography with biliary stent placement should be considered in cases with obstructive jaundice.

## INTRODUCTION

Endoscopic ultrasound‐guided fine needle aspiration (EUS‐FNA) is a safe and effective tool for the pathological diagnosis of solid pancreatic lesions (SPLs).^1^, ^2^, ^3^, ^4^ However, the diagnostic yield of EUS‐FNA is influenced by several factors, such as the size of SPLs, type of needle, and availability of rapid on‐site evaluation (ROSE).^3^, ^5^, ^6^ Meanwhile, patients with SPLs in the head of the pancreas often present with obstructive jaundice and endoscopic retrograde cholangiopancreatography (ERCP) is performed for biliary drainage as well as transpapillary tissue sampling, but it is well known that sensitivity of transpapillary sampling of biliary stricture is relatively low.^7^, ^8^

In cases with indwelling biliary stents, inflammation of the bile duct, pneumobilia, and acoustic shadow might impair the EUS image and affect the performance of EUS‐FNA^9^, ^10^, ^11^, ^12^ and several studies have addressed the impact of biliary stents on EUS‐FNA for SPLs.^9^, ^10^, ^11^, ^13^, ^14^, ^15^, ^16^, ^17^ However, the study results are conflicting and the diagnostic yield might differ according to the study cohort such as the location of SPLs and the ratio of malignancy.

To address this issue, we retrospectively studied the diagnostic yield of EUS‐FNA for SPLs with or without biliary stents in our center and further conducted a meta‐analysis including our study results to evaluate the impact of biliary stents on EUS‐FNA as well as subgroup analyses of SPLs in the head of the pancreas.

## METHODS

### Patients

This is a single‐center, retrospective study at the University of Tokyo Hospital. Data on consecutive patients who underwent EUS‐FNA between January 2017 and December 2021 for SPLs were retrieved from our prospectively maintained database at the University of Tokyo Hospital. Inclusion criteria were patients aged ≥18 years who underwent EUS‐FNA for evaluation of SPLs and whose cytological and histological analyses were available. Exclusion criteria were patients with a pancreatic cystic lesion or an extra‐pancreatic lesion. This study was conducted according to the guidelines in the Helsinki Declaration and was approved by the ethics committee of the University of Tokyo. Informed consent was obtained from the participants on an opt‐out basis given the retrospective nature of the study.

### EUS‐FNA procedure

All EUS procedures were performed using a curved linear array echoendoscope (GF‐UCT260; Olympus Medical Systems, Tokyo, Japan or EG‐580‐UT; Fujifilm Medical Systems, Tokyo, Japan), which was connected to a processor featuring color Doppler function (EU‐ME2; Olympus Medical Systems or SU‐1, Fujifilm Medical Systems) under moderate sedation. Antithrombotic agents were managed according to the guidelines for gastroenterological endoscopy by the Japan Gastroenterological Endoscopy Society.^18^ EUS‐guided tissue acquisition was performed using FNA needles (Expect; Boston Scientific Japan, Tokyo, Japan; EZ shot3 Plus; Olympus Corporation, Tokyo, Japan) and/or fine needle biopsy (FNB) needles (Acquire; Boston Scientific Japan, EchoTip Procore; Cook Medical Japan G.K., Tokyo, Japan, SharkCore; Medtronic Japan Corporation, Tokyo, Japan, SonoTip TopGain; Medi‐Globe GmbH, Rosenheim, Germany). The needle type and application of suction were decided at the discretion of endoscopists, and EUS‐FNA was repeated until enough visible core tissue was obtained macroscopically. Rapid on‐site evaluation was performed when available.

### Cytological and histological diagnosis

The cytological and histological diagnoses were made by expert pathologists. Samples for cytology were graded into one of five categories (I: benign, II: atypical but no evidence of malignancy, III: suggestive of, but not conclusive for malignancy, IV: suspicious for malignancy, V: malignant). Samples reported as malignant or suspicious for malignancy either by cytology or histology were classified as positive for malignancy.

The final diagnosis was based on histology obtained by surgical resection or on unequivocal EUS‐FNA results in nonsurgical patients. Nonsurgical patients with nondiagnostic EUS‐FNA, whose clinical and radiological course was consistent with malignancy, were considered malignant. The diagnosis of benign diseases required a follow‐up of at least 6 months with compatible clinical and radiological courses.

### Outcome measures

The primary endpoint is the accuracy of EUS‐FNA for SPLs with or without biliary stents. Secondary endpoints are the sensitivity and specificity of EUS‐FNA for SPLs and prognostic factors to affect the diagnostic yield of EUS‐FNA.

### Meta‐analysis of the diagnostic yield of EUS‐FNA

We also conducted a meta‐analysis following PRISMA guidelines and compared the diagnostic yield of EUS‐FNA for SPLs with or without biliary stents. The MEDLINE/PubMed and Web of Science were searched until March 2022. Two researchers (Go Endo and Kazunaga Ishigaki) independently screened the titles and abstracts of all initially identified studies. Disagreements were resolved through discussion with another researcher (Tsuyoshi Hamada). The key search words were “Endoscopic ultrasound,” “EUS‐FNA,” “EUS‐FNB,” “needle aspiration,” “needle biopsy,” “pancreatic neoplasms,” and “stent”. The search was limited to fully published articles in English.

### Statistical analysis

Continuous variables were presented as the median and range and categorical variables as the number and percentage. Statistical comparisons were performed with Wilcoxon's rank sum test for continuous variables and Fisher's exact test for categorical variables. Logistic regression analyses were performed to identify factors associated with diagnostic accuracy. In addition to the known factors affecting the diagnostic yield of EUS‐FNA such as the location and the size of SPLs, needle type, and number of passes,^5^, ^6^, ^9^, ^10^, ^16^ factors with *p* ≤ 0.10 in the univariate analyses were included in the multivariate logistic regression analyses. A *p*‐value of < 0.05 in a two‐tailed test was considered a statistically significant difference. All statistical analyses were performed using Stata version 17.0 (StataCorp, College Station, Texas, USA) or Review Manager 5.4.1 (RevMan; https://revman.cochrane.org/).

This study was conducted in accordance with the Declaration of Helsinki. The local ethical committee approved this study (No. 2058).

## RESULTS

### Patient characteristics and final diagnosis

Patient Characteristics are shown in Table 1. A total of 509 patients with SPLs were included in this study. The median age of patients was 70 (25–91) years. Of 509 patients, 17 patients had two lesions and two patients had three lesions. ERCP for obstructive jaundice was performed first for biliary stent placement and pathological diagnosis prior to EUS‐FNA in 40 patients. Of 40 patients, 38 had a plastic stent (PS) and two had a self‐expandable metallic stent (SEMS). Details of the final diagnosis are shown in Table 2. The final diagnosis was pancreatic ductal adenocarcinoma in 57%, autoimmune pancreatitis in 14%, neuroendocrine tumor in 9%, and metastatic lesion in 2%.

**TABLE 1:** Patient characteristics, final diagnosis, and details of endoscopic ultrasound‐guided fine needle aspiration.

	Overall (*n* = 530)	With biliary stents (*n* = 40)	Without biliary stents (*n* = 490)	*p‐*Value
**Patient characteristics**				
Age, years	70 (25‐91)	70.5 (38–85)	69.5 (25–91)	0.42
Gender, male	349 (65.9)	29 (72.5)	320 (65.3)	0.23
Biliary stent	40 (7.5)			
Plastic stent	38 (95.0)			
Metal stent	2 (5.0)			
Pancreatic stent	13 (2.5)	5 (12.5)	8 (1.63)	<0.01
**Final diagnosis**				
Malignant	370 (69.8)	31 (77.5)	339 (69.2)	0.18
Benign	160 (30.2)	9 (22.5)	151 (30.8)	0.18
**Details of EUS‐FNA**				
Procedure time, min	40 (10‐115)	38.5 (22‐85)	40 (15–115)	0.41
Location of the lesion				
Head	213 (40.2)	34 (85.0)	179 (36.5)	<0.01
Body	207 (39.1)	5 (12.5)	202 (41.2)	<0.01
Tail	110 (20.8)	1 (2.5)	109 (22.2)	<0.01
Size of the lesion, mm	22 (3‐70)	26 (12–70)	21 (3–66)	0.30
**Puncture route**				
Stomach	305 (57.6)	4 (10.0)	301 (61.4)	<0.01
Duodenum	219 (41.3)	36 (90.0)	183 (37.4)	<0.01
Jejunum	6 (1.0)	0	6 (1.2)	0.10
**Needle size**				
19G	8 (1.4)	0	8 (1.6)	0.19
20G	24 (4.3)	2 (4.1)	22 (4.3)	0.38
22G	459 (82.0)	35 (71.4)	424 (83.0)	0.42
25G	69 (12.3)	12 (24.5)	57 (11.2)	0.37
**Needle type**				
FNA	136 (24.3)	13 (26.5)	123 (24.1)	0.38
FNB	424 (75.7)	36 (73.5)	388 (76.0)	0.38
**Suction method**				
Suction	484 (86.4)	42 (85.7)	442 (86.5)	0.44
No suction	31 (5.5)	0	31 (6.0)	0.14
Other	45 (8.0)	7 (14.3)	38 (7.4)	0.21
Number of passes	4 (1–8)	4 (2–8)	4 (1‐8)	0.18
Rapid on‐site evaluation	11 (2.1)	1 (2.5)	10 (2.0)	0.58

Numbers are shown in *n* (%) or median (range).

Abbreviations: EUS, endoscopic ultrasound; FNA, fine needle aspiration; FNB, fine needle biopsy.

**TABLE 2:** Details of the final diagnosis.

**Malignant**	370 (69.8)
Adenocarcinoma	300 (56.6)
Neuroendocrine tumor	45 (8.5)
Metastatic lesion	12 (2.3)
Lymphoma	5 (0.9)
Liposarcoma	3 (0.6)
Acinar cell carcinoma	2 (0.4)
Adenosquamous carcinoma	2 (0.4)
Anaplastic carcinoma	1 (0.2)
**Benign**	160 (30.2)
Autoimmune pancreatitis	75 (14.2)
Mass‐forming pancreatitis	52 (9.8)
Chronic pancreatitis	22 (4.2)
Solid pseudopapillary neoplasm	4 (0.8)
Accessory spleen	4 (0.8)
Schwannoma	2 (0.4)
Lipoma	1 (1.3)

Numbers are shown in *n* (%)

### EUS‐FNA procedures

Details of EUS‐FNA are shown in Table 1. SPLs were located in the head in 40%. The median size of the lesion was 22 (3‐70) mm. The puncture was transgastric in 305 passes (58%). The needle size was 22‐gauge in 82% and the FNB needle was used in 76%. The procedures were performed with suction in 484 cases (91%), without suction in 31 cases (6%), and both in 11 cases (2%). The median number of passes was 4 (1–8). Rapid on‐site evaluation was performed in 11 (2%) patients.

### Diagnostic performance

The diagnostic performance of EUS‐FNA for SPLs is presented in Table 3. Overall accuracy, sensitivity, and specificity were 93.2%, 90.0%, and 100%, respectively. Accuracy was lower in patients with biliary stents: 77.5% and 94.5% in patients with stents and without stents groups (*p* < 0.01). Sensitivity was also lower in patients with biliary stents: 71.0% and 91.7% in with stents and without stents groups (*p* < 0.01). No differences were observed in specificity between the two groups.

**TABLE 3:** Diagnostic performance of endoscopic ultrasound‐guided fine needle aspiration for solid pancreatic lesions in patients with and without biliary stents.

	Overall (*n* = 530)	With biliary stents (*n* = 40)	Without biliary stents (*n* = 490)	*p‐*Value
True positive, *n*	333	22	311	0.31
True negative, *n*	160	9	151	0.37
False positive, *n*	0	0	0	―
False negative, *n*	37	9	28	<0.01
Accuracy (95% CI)	93.2% (91.0%–95.4%)	77.5% (64.0%–91.0%)	94.5% (92.5%–96.5%)	<0.01
Sensitivity (95% CI)	90.0% (86.9%–93.1%)	71.0% (54.0%–87.9%)	91.7% (88.8%–94.7%)	<0.01
Specificity (95% CI)	100% (97.7%–100%)	100% (66.4%–100%)	100% (97.6%–100%)	―

Abbreviation: CI, confidence interval.

Univariate and multivariate logistic regression analyses for diagnostic accuracy in the overall population are presented in Table 4. In the univariate analyses, puncture from the duodenum or jejunum (89.8% vs 95.7%, *p* < 0.01) and the presence of biliary stents (77.5% vs 94.5%, *p* < 0.01) were associated with the low accuracy of EUS‐FNA. In the multivariate analysis, age over 70 years (odds ratio [OR] of 0.43, 95% confidence interval [CI] 0.20–0.90, *p* = 0.03), located in the pancreatic head (OR of 0.30, 95% CI 0.10–0.91, *p* = 0.03), tumor size < 20 mm (OR of 0.35, 95% CI 0.16–0.77, *p* = 0.01), puncture from duodenum or jejunum (OR of 0.16, 95% CI 0.05–0.51, *p* < 0.01), and presence of biliary stents (OR of 0.20, 95% CI 0.08–0.53, *p* < 0.01) were associated with the low accuracy of EUS‐FNA.

**TABLE 4:** Univariate and multivariate logistic regression of factors associated with the accuracy of endoscopic ultrasound‐guided fine needle aspiration.

	Univariate analysis			Multivariate analysis	
	*n*	Accuracy	*p‐*Value	OR (95% CI)	*p‐*Value
Age					
<70 years	263	95.4%	0.06		
≥70 years	267	91.0%		0.43 (0.20–0.90)	0.03
Gender					
Male	349	93.1%	0.99		
Female	181	93.4%			
Location of the lesion					
Head	213	91.6%	0.22	0.30 (0.10–0.91)	0.03
Body/tail	317	94.3%			
Size of the lesion					
<20 mm	190	90.5%	0.07	0.35 (0.16–0.77)	0.01
≥20 mm	340	94.7%			
Puncture route					
Stomach	305	95.7%	<0.01		
Duodenum/Jejunum	225	89.8%		0.16 (0.05–0.51)	< 0.01
**Needle size**					
19G/20G	32	96.9%	0.72		
22G/25G	528	93.0%			
**Needle type**					
FNA	136	90.8%	0.30	1.00 (0.44–2.29)	0.99
FNB	424	93.9%			
**Number of passes**					
<5	420	93.8%	0.29		
≥5	110	90.9%		0.71 (0.32–1.59)	0.41
ROSE					
Yes	11	100%	0.91		
No	519	93.1%			
Biliary stent					
Yes	40	77.5%	<0.01	0.20 (0.08–0.53)	< 0.01
No	490	94.5%			
Pancreatic stent					
Yes	13	92.3%	0.60		
No	517	93.2%			

Abbreviations: CI, confidence interval; FNA, fine needle aspiration; FNB, fine needle biopsy; G, gauge; OR, odds ratio; ROSE, rapid on‐site evaluation.

Furthermore, a subgroup analysis of SPLs located in the head was performed (Table 5). Accuracy was lower in patients with biliary stents: 79.4% and 93.9% in patients with stents and without stents groups (*p* = 0.01). Sensitivity was also lower in patients with biliary stents: 74.1% and 91.5% in with stents and without stents groups (*p* = 0.02). No differences were observed for specificity between with stents and without‐stents groups.

**TABLE 5:** Diagnostic performance of endoscopic ultrasound‐guided fine needle aspiration for solid pancreatic head lesions in patients with or without biliary stents.

	Overall (*n* = 213)	With biliary stents (*n* = 34)	Without biliary stents (*n* = 179)	*p‐*Value
True positive, *n*	139	20	119	0.43
True negative, *n*	56	7	49	0.53
False positive, *n*	0	0	0	―
False negative, *n*	18	7	11	0.01
Accuracy (95%CI)	91.5% (87.8%–95.3%)	79.4% (65.1%–93.7%)	93.9% (90.3%‐97.4%)‐	0.01
Sensitivity (95%CI)	88.5% (83.5%–93.6%)	74.1% (56.4%–91.7%)	91.5% (86.7%–96.4%)	0.02
Specificity (95%CI)	100% (93.6%–100%)	100% (59.0%–100%)	100% (92.7%–100%)	―

Abbreviation: CI, confidence interval.

### Meta‐analysis of EUS‐FNA with or without biliary stents

**FIGURE 1:**
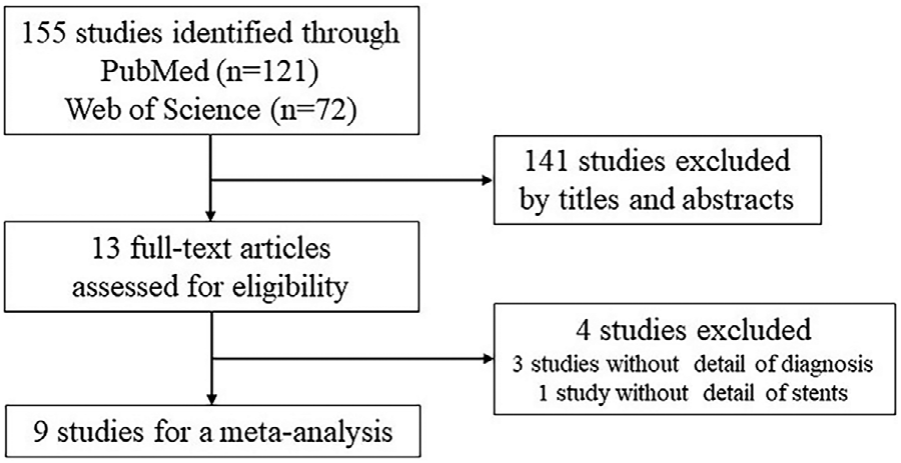
Flowchart of the study selection for meta‐analysis.

We identified eight studies analyzing the diagnostic yield of EUS‐FNA with and without biliary stents,^9^, ^10^, ^11^, ^13^, ^14^, ^15^, ^16^, ^17^ and one study analyzing with PS and SEMS^19^ (Figure 1). The details of nine studies are shown in Table 6.

**TABLE 6:** Study details.

Author	Year	*N*	Study design	Inclusion criteria	Final diagnosis	Biliary stents	Needle type
Fisher^13^	2011	268	Retrospective	Cancer in HOP	268 malignant (100%)	98 PS 170 without stents	FNA
Ranney^14^	2012	214	Retrospective	SPLs with jaundice	197 malignant (92.1%)	105 PS, 45 SEMS 64 without stents	FNA
Siddiqui^19^	2012	677	Retrospective	SPLs in HOP with jaundice	620 malignant (91.6%)	577 PS, 100 SEMS 0 without stents	FNA
Kim^9^	2015	180	Retrospective	SPLs with jaundice	172 malignant (95.6%)	64 PS, 11 SEMS 105 without stents	75 FNA 105 FNA and FNB
Seicean^15^	2016	118	Prospective	SPLs	107 malignant (90.7%)	11 PS 107 without stents	FNA
Antonini^16^	2017	130	Retrospective	SPLs in HOP with jaundice	125 malignant (96.2%)	56 PS 74 without stents	FNB
Bekkali^10^	2019	698	Retrospective	SPLs in HOP	581 malignant (83.2%)	167 PS, 163 SEMS 368 without stents	290 FNA 408 FNB
Crino^11^	2021	842	Retrospective	SPLs in HOP with jaundice	816 malignant (97.0%)	217 PS, 130 SEMS 495 without stents	FNB
Constantinescu^17^	2022	243	Retrospective	Pancreatic cancer	243 malignant (100%)	58 PS, 10 SEMS 175 without stents	FNA/FNB (unknown)
Our study	2022	530	Retrospective	SPLs	370 malignant (69.8%)	38 PS, two SEMS 490 without stents	106 FNA 30 FNA and FNB 394 FNB

Abbreviations: FNA, fine needle aspiration; FNB, fine needle biopsy; HOP, head of pancreas; PS, plastic stent; SEMS, self‐expandable metallic stent; SPLs, solid pancreatic lesions.

**FIGURE 2:**
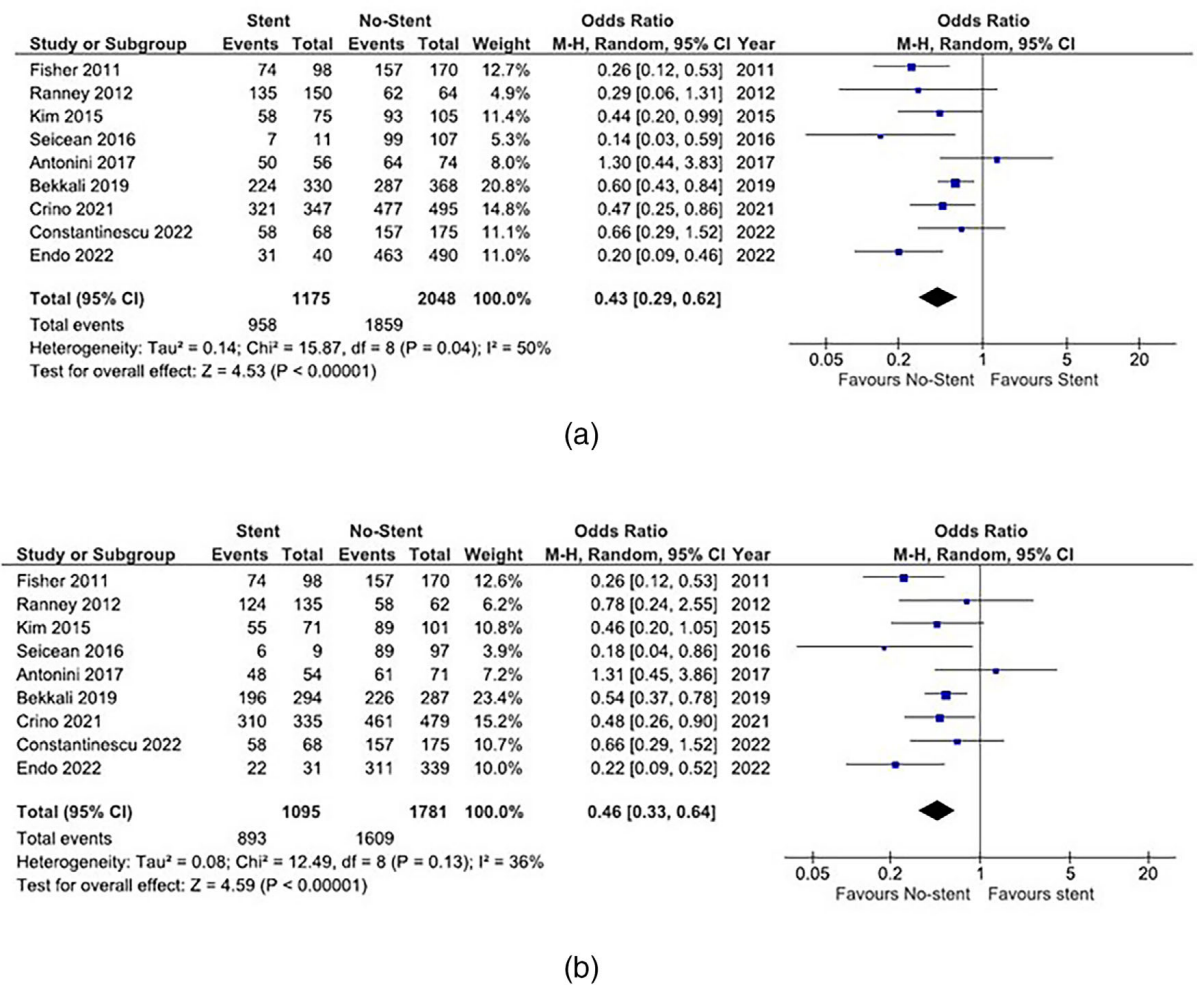
Comparison of diagnostic yield of endoscopic ultrasound‐guided fine needle aspiration (EUS‐FNA) with biliary stents and without biliary stents. The odds ratio (OR) is presented for each study (center of the square) with a 95% confidence interval (CI; horizontal line). Summary OR based on a meta‐analysis via the random‐effect model is presented at the bottom (center of the diamond) with 95% CI (the width of the diamond). The *p*‐value for the Q‐statistic for between‐study heterogeneity is shown. a. Comparison of accuracy. b. Comparison of sensitivity.

**FIGURE 3:**
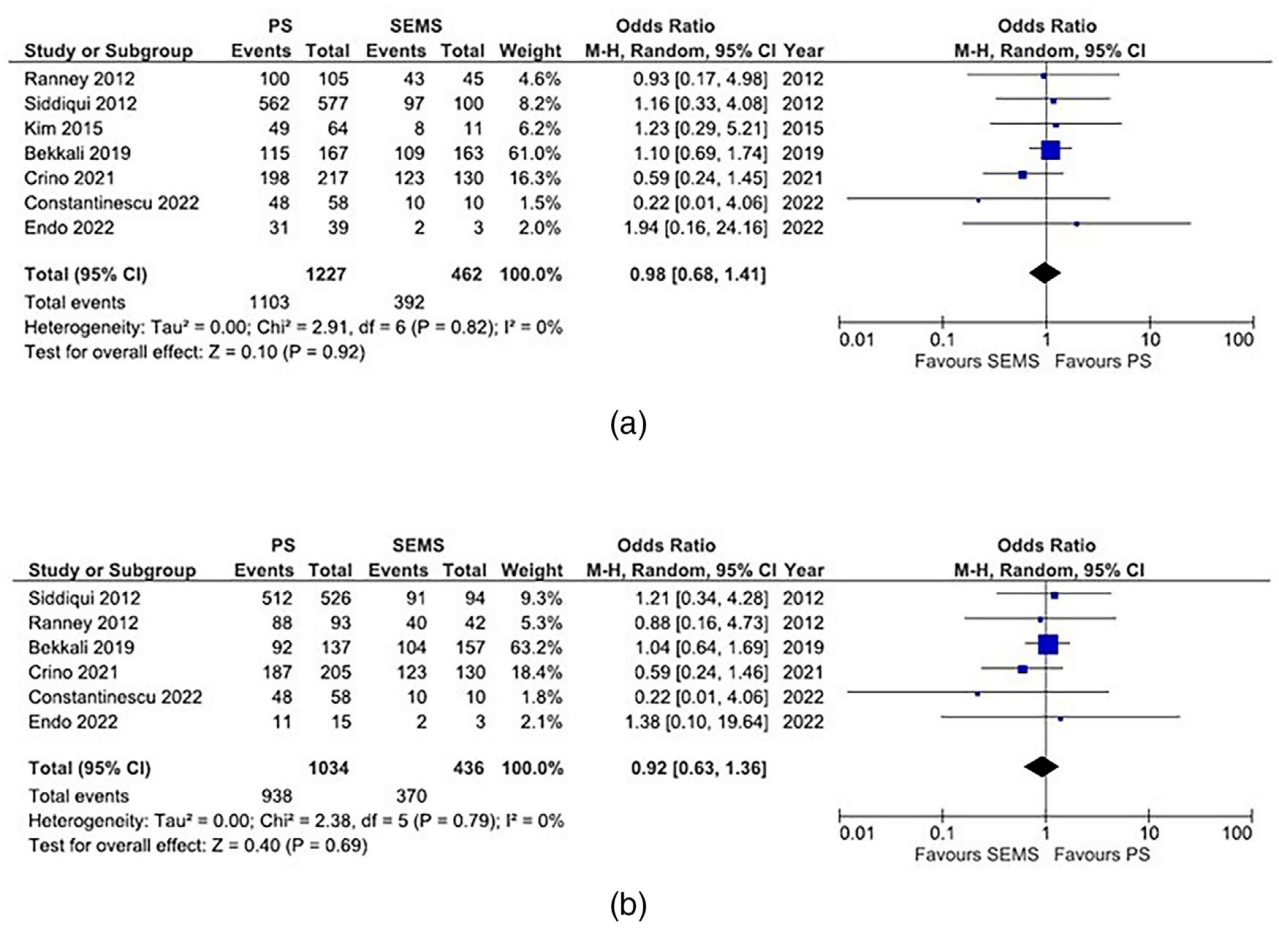
Comparison of diagnostic yield of endoscopic ultrasound‐guided fine needle aspiration (EUS‐FNA) between PS and SEMS. The odds ratio (OR) is presented for each study (center of the square) with a 95% confidence interval (CI; horizontal line). Summary OR based on a meta‐analysis via the random‐effect model is presented at the bottom (center of the diamond) with 95% CI (the width of the diamond). The *p*‐value for the Q‐statistic for between‐study heterogeneity is shown. a. Comparison of accuracy. b. Comparison of sensitivity.

We conducted a meta‐analysis including our data, comparing the accuracy and sensitivity of EUS‐FNA in patients with biliary stents and without biliary stents (Figure 2 and Figure S1). Accuracy was lower in patients with biliary stents than in those without biliary stents (OR of 0.43, 95% CI 0.29–0.62, *p* < 0.01). Sensitivity was also lower in patients with biliary stents (OR of 0.46, 95% CI 0.33‐0.64, *p* < 0.01). A meta‐analysis comparing the accuracy and sensitivity of EUS‐FNA in patients with PS and SEMS was also conducted (Figure 3). No significant differences were observed for accuracy (OR of 0.98, 95% CI 0.68–1.41, *p* = 0.92) and sensitivity (OR of 0.92, 95% CI 0.63–1.36, *p* = 0.69) according to the stent type.

**FIGURE 4:**
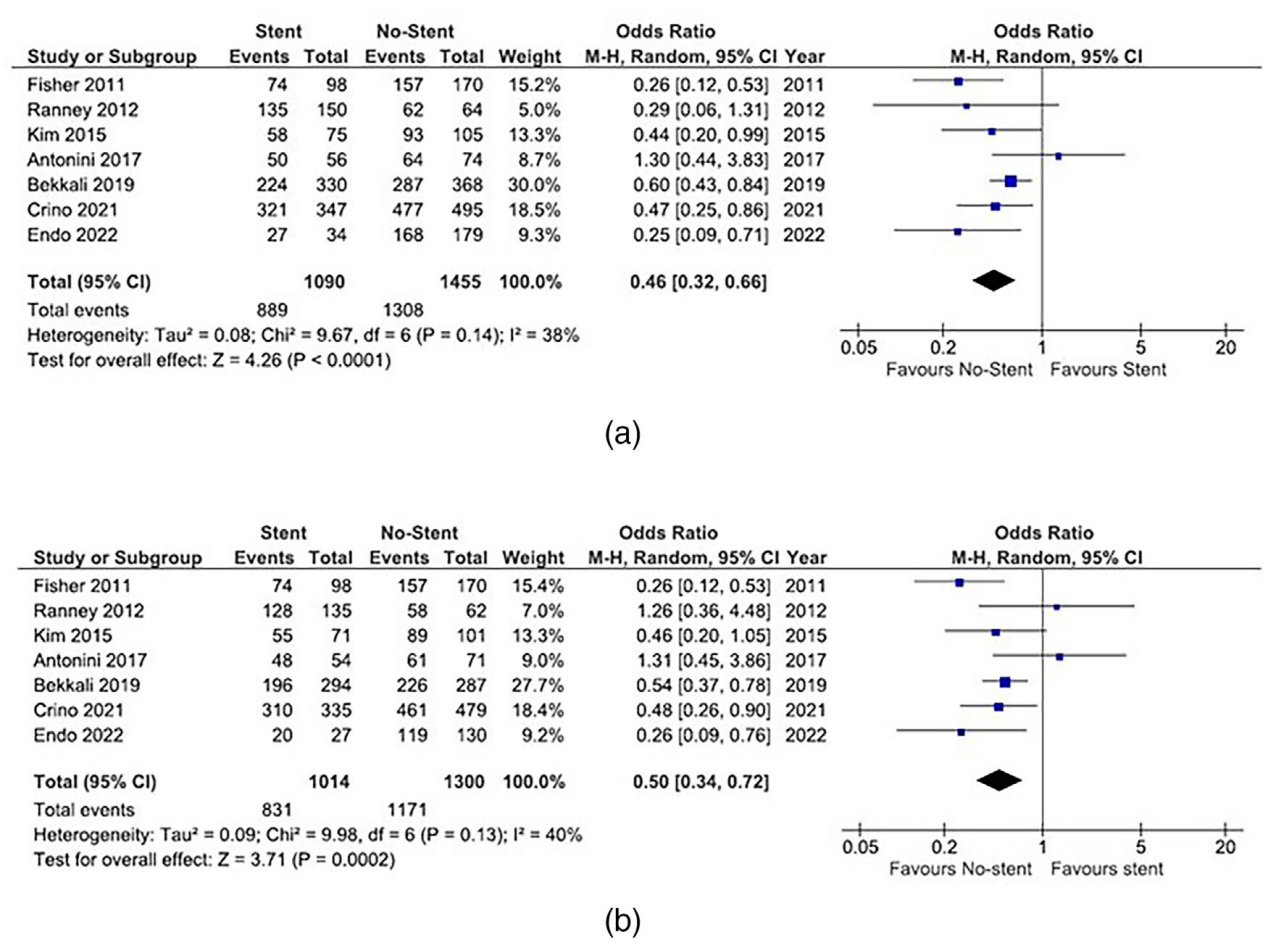
Subgroup analysis of solid pancreatic head lesions. Comparison of diagnostic yield of endoscopic ultrasound‐guided fine needle aspiration (EUS‐FNA) with biliary stents and without biliary stents. The odds ratio (OR) is presented for each study (center of the square) with a 95% confidence interval (CI; horizontal line). Summary OR based on a meta‐analysis via the random‐effect model is presented at the bottom (center of the diamond) with 95% CI (the width of the diamond). The p‐value for the Q‐statistic for between‐study heterogeneity is shown. a. Comparison of accuracy. b. Comparison of sensitivity.

A subgroup analysis of SPLs in the head was conducted (Figure 4), which confirmed lower diagnostic yield in patients with biliary stents, similar to the overall cohort. Accuracy was lower in patients with biliary stents than in those without biliary stents (OR of 0.46, 95% CI 0.32–0.66, *p* < 0.01). Sensitivity was also lower in patients with biliary stents (OR of 0.50, 95% CI 0.34–0.72, *p* < 0.01).

## DISCUSSIONS

In our single‐center retrospective study, the presence of biliary stents had a negative impact on the diagnostic yield of EUS‐FNA, including accuracy (77.5% and 94.5% in patients with stents and without stents, *p* < 0.01) and sensitivity (71.0% and 91.7% in patients with stents and without stents, *p* < 0.01). Among cases with indwelling biliary stents, the type of stents (SEMS or PSs) did not affect the diagnostic yield of EUS‐FNA. A meta‐analysis of 3, 860 EUS‐FNA, including our data, was in consistency with our single‐center study results.

In patients with distal biliary stricture, while international consensus statements^8^ recommend ERCP‐guided tissue sampling should be first attempted when biliary drainage is required, another proposal^20^ recommends EUS‐FNA first prior to ERCP. However, the sensitivity of transpapillary tissue sampling of biliary stricture is low,^7^ and given the negative impact of indwelling biliary stents on the diagnostic yield of EUS‐FNA in our analysis, EUS‐FNA first approach prior to ERCP would be recommended for tissue sampling of SPLs with obstructive jaundice. However, we should consider the nature of biliary strictures, extrinsic or intrinsic. As Lee et al.^21^ reported the diagnostic yield of transpapillary biopsy of extrinsic biliary structure due to SPLs is lower than intrinsic biliary stricture, EUS‐FNA should be performed for extrinsic biliary stricture on EUS, and ERCP with transpapillary sampling can be primarily performed for intrinsic stricture due to cholangiocarcinoma.

Since indwelling biliary stents would impair EUS images by artifacts of acoustic shadows or reverberation, a large diameter SEMS can potentially affect the diagnostic yield of EUS‐FNA more than PSs. Bekkali et al.^10^ reported that SEMS was associated with the necessity of repeat procedures compared to no stents or PSs. However, the type of stents did not affect the diagnostic yield in our meta‐analysis. Needle selection in cases with biliary stents needs to be discussed, too. Although the type of needle, FNA or FNB did not affect the diagnostic yield in our cohort, two studies^9^, ^10^ reported the use of FNB needles was associated with better accuracy. Thus, FNB needles might be preferred if EUS‐FNA is to be performed in cases with biliary stents for some reason.

Our study has several limitations. First, there is considerable heterogeneity among studies included in the meta‐analysis. For example, the definition of malignancy was not uniform; while some studies classified suspicious malignancy as malignant, others as non‐malignant. In addition, some studies included only SPLs in the head of the pancreas. Secondly, the number of cases with SEMS was small and the impact of SEMS compared to PSs on diagnostic performance of EUS‐FNA might be underestimated. Third, the safety and cost of EUS‐FNA were not included in our analysis. Despite its low adverse event rate, EUS‐FNA prior to ERCP may predispose patients to the risk of pancreatitis, hemobilia, or tumor seeding.^4^ Although a single session EUS and ERCP is reportedly feasible,^22^ its cost‐effectiveness should be further investigated.

In conclusion, the presence of biliary stents had a negative impact on the diagnostic performance of EUS‐FNA, and EUS‐FNA prior to ERCP for biliary stent placement should be considered in cases with obstructive jaundice due to SPLs.

## CONFLICT OF INTEREST STATEMENT

Author Mitsuhiro Fujishiro has received honoraria from Fujifilm Corporation and Olympus Corporation and research grants from Fujifilm Corporation and Olympus Corporation. Author Yousuke Nakai is an editorial board member of Digestive Endoscopy and has received honoraria from Boston Scientific Japan, Gadelius medical corporation, Olympus Corporation, and research grants from Fujifilm Corporation and HOYA Corporation.

## Supporting information


Supplemental Figure S1

**Supplemental Figure S1**. Funnel plots to examine potential publication bias in odds ratio. The x‐axis represents odds ratio, and the y‐axis displays the standard error of log (odds ratio). a. Comparison of accuracy with and without biliary stents. b. Comparison of sensitivity with and without biliary stents. c. Comparison of accuracy between PS and SEMS. d. Comparison of sensitivity between PS and SEMS. e. Comparison of accuracy of EUS‐FNA for solid pancreatic head lesions with and without biliary stents. f. Comparison of sensitivity of EUS‐FNA for solid pancreatic head lesions with and without biliary stents.
