# Supplementary figures and images for: The impact of biliary stents on the diagnostic yield of endoscopic ultrasound‐guided fine needle aspiration for solid pancreatic lesions: A single‐center retrospective study and meta‐analysis
Source: DEN Open. 2023 Jul 10;4(1):e250. doi: 10.1002/deo2.250 (PMC10333724; doi:10.1002/deo2.250)

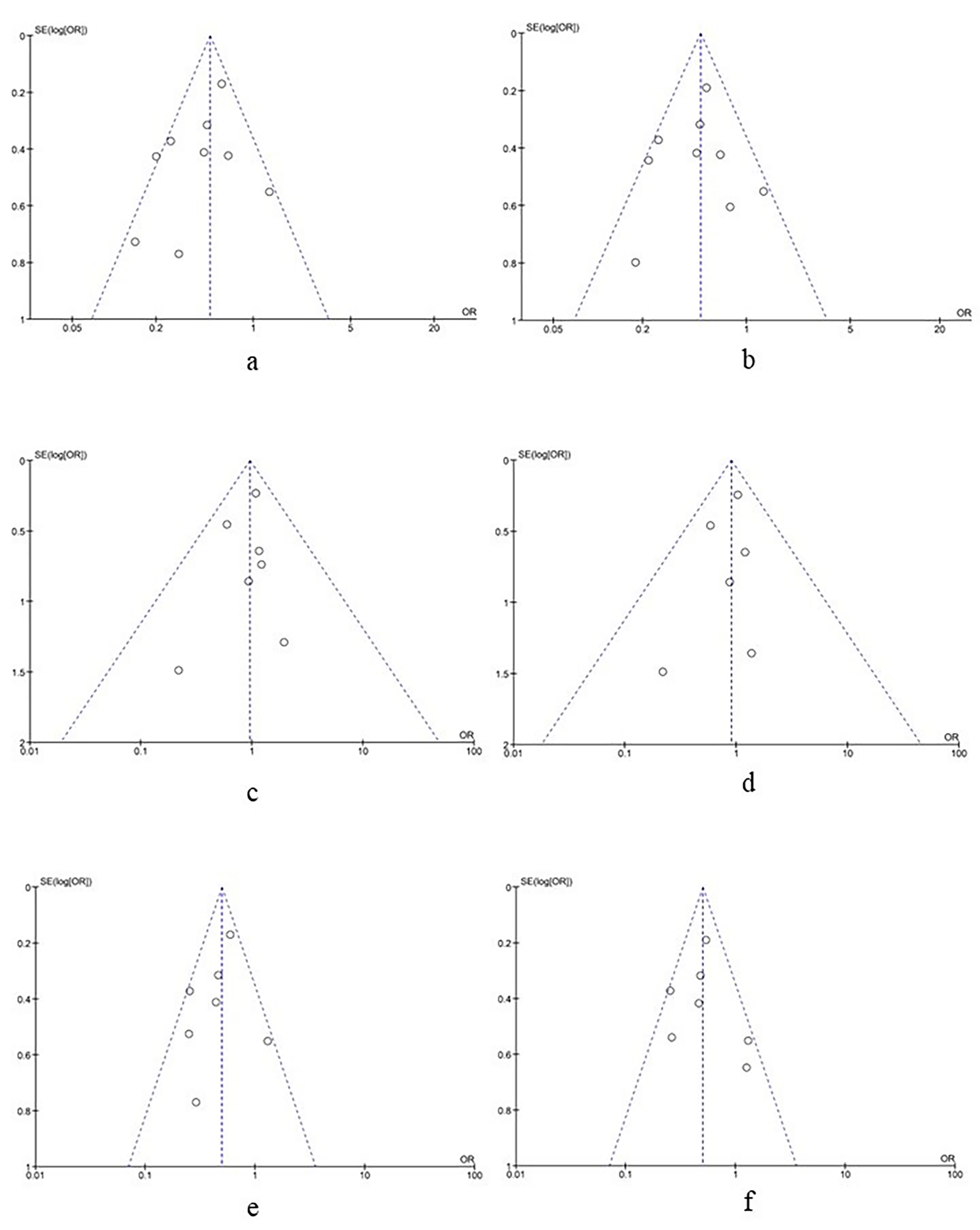

Supplement: Supplementary file 1 — Supplemental Figure S1 [file DEO2-4-e250-s002.tif]
