# Supplementary material for: The impact of biliary stents on the diagnostic yield of endoscopic ultrasound‐guided fine needle aspiration for solid pancreatic lesions: A single‐center retrospective study and meta‐analysis
Source: DEN Open. 2023 Jul 10;4(1):e250. doi: 10.1002/deo2.250 (PMC10333724; doi:10.1002/deo2.250)
Supplement: Supplementary file 2 — Supplemental Figure S1. Funnel plots to examine potential publication bias in odds ratio. The x‐axis represents odds ratio, and the y‐axis displays the standard error of log (odds ratio). a. Comparison of accuracy with and without biliary stents. b. Comparison of sensitivity with and without biliary stents. c. Comparison of accuracy between PS and SEMS. d. Comparison of sensitivity between PS and SEMS. e. Comparison of accuracy of EUS‐FNA for solid pancreatic head lesions with and without biliary stents. f. Comparison of sensitivity of EUS‐FNA for solid pancreatic head lesions with and without biliary stents. [file DEO2-4-e250-s001.docx]

**Supplementary material**

**Supplemental Figure S1.**

Funnel plots to examine potential publication bias in odds ratio. The x-axis represents odds ratio, and the y-axis displays the standard error of log (odds ratio).

a. Comparison of accuracy with and without biliary stents. b. Comparison of sensitivity with and without biliary stents. c. Comparison of accuracy between PS and SEMS. d. Comparison of sensitivity between PS and SEMS. e. Comparison of accuracy of EUS-FNA for solid pancreatic head lesions with and without biliary stents. f. Comparison of sensitivity of EUS-FNA for solid pancreatic head lesions with and without biliary stents.
